# Medical Student Experiences of Uncertainty Tolerance Moderators: A Longitudinal Qualitative Study

**DOI:** 10.3389/fmed.2022.864141

**Published:** 2022-04-25

**Authors:** Georgina C. Stephens, Mahbub Sarkar, Michelle D. Lazarus

**Affiliations:** ^1^Centre for Human Anatomy Education, Department of Anatomy and Developmental Biology, Monash University, Melbourne, VIC, Australia; ^2^Monash Centre for Scholarship in Health Education, Faculty of Medicine, Nursing and Health Sciences, Monash University, Melbourne, VIC, Australia

**Keywords:** uncertainty, tolerance, moderator, medical student, medical education, qualitative

## Abstract

**Introduction:**

Uncertainty tolerance (UT), a construct explicating individuals' response to perceived uncertainty, is increasingly considered a competency for effective medical practice. Lower UT among physicians is linked with negative outcomes, including less favorable attitudes toward patient-centered care, and increased burnout risk. Despite decades of research, as yet few have engaged methodological approaches aiming to understand the factors that may influence medical students' UT (so-called moderators). Such knowledge, though, could inform teaching practices for fostering learners' skills for managing uncertainties. Accordingly, we asked “*What factors do medical students in their clinical years perceive as moderating their perceptions of, and responses to, uncertainty?”*

**Methods:**

We conducted a qualitative study with forty-one medical students in clinical years at an Australian medical school, with data collected throughout 2020. Participants described their experiences of uncertainty through both in-semester reflective diary entries (*n* = 230) and end of semester group or individual semi-structured interviews (*n* = 40). Data were analyzed using a team-based framework analysis approach.

**Results:**

Four major themes of UT moderators were identified: (1) Individual factors, (2) Sociocultural factors, (3) Academic factors and (4) Reflective learning. Aspects of individual, sociocultural and academic factors were perceived as having either positive or negative influences on students' perceptions of uncertainty. By contrast, reflective learning was described as having a predominantly positive influence on students' perceptions of uncertainty, with students noting learning opportunities and personal growth afforded through uncertain experiences.

**Conclusions:**

As healthcare becomes increasingly complex, a future challenge is equipping our medical students with strategies and skills to manage uncertainties. Our study identified multiple moderators of medical students' UT, key among them being reflective learning. We also identified UT moderators that contemporary and future medical educators may be able to harness in order to develop learner UT as a healthcare graduate attribute, especially through teaching practices such as intellectual candor. Further research is now required to evaluate the impact of proposed educational interventions, and to develop effective assessments of students' skills for managing clinical uncertainties.

## Introduction

Despite significant advances in medical knowledge and evidence-based practice throughout the 20th century, uncertainty remains an inherent and pervasive aspect of healthcare practice (1). Recently, the COVID-19 pandemic has further underscored the potential magnitude and importance of healthcare-related uncertainty, and the need for healthcare professionals to possess skills for managing these implicit uncertainties (2). Uncertainty tolerance (UT), a construct encompassing how individuals perceive and respond to uncertainty across their cognitions, emotions and behaviors (3), is therefore increasingly considered a necessary attribute for medical graduates (1, 4, 5).

UT may be considered a future protecting skill for healthcare professionals, as advances in medical technology supersede human data processing abilities. For example, healthcare is increasingly engaging artificial intelligence (AI) in applications such as diagnosis, decision making and patient education (6, 7). AI functions in a realm of pattern recognition, categorization and precision (7), and has been found to have limited capacity to tolerate uncertainty (8, 9). Healthcare is, however, typically complex and ambiguous, and may not be easily reduced to simple categories (10). Thus, to prepare for this technology-laden healthcare landscape, future healthcare professionals may benefit from developing UT skills in order to better interrogate AI outputs for potential uncertainties, and facilitate working alongside AI.

Presently, research demonstrates important links between physician and medical student UT with healthcare-related outcomes (11). Lower UT is associated with negative outcomes such as increased healthcare resource use and more paternalistic patient care attitudes (11–13), whereas higher UT appears to be protective against declining attitudes toward underserved patient populations (14). The clearest association between physician and medical student UT is, however, with their own psychological wellbeing, with lower UT associated with higher rates of psychological distress and risk of burnout (11, 15). Within the uncertainties of the pandemic context and reports of largescale healthcare worker burnout and resignation (16), better understanding medical students' UT, and how this can be developed as a graduate attribute, is timely.

The integrative model by Hillen et al. (3) provides a contemporary and wide-ranging conceptual framework for researching UT. Within the model, a *stimulus* is the underlying source of uncertainty, and is defined in terms of ambiguity, probability and complexity. Thus, terms such as “tolerance for ambiguity”, which are also used within medical education research, may be considered subordinate to the UT construct. Hillen et al. (3) do highlight, however, that clear conceptual differences between UT and tolerance for ambiguity were unable to be identified.

Following *perception* of uncertainty, an individual *appraises* and *responds* to uncertainty across cognitive, emotional and behavioral response domains. *Moderators* may then act to influence either the perception of or the responses to uncertainty, and are categorized as (a) stimulus characteristics, (b) individual characteristics, (c) situational characteristics, (d) cultural factors, and (e) social factors, but are not further defined by Hillen et al. (3). The inclusion of moderators within the model aligns with recent research supportive that UT is (at least in part) a modifiable state, whereas early research typically conceptualized UT as an immutable personality trait (17, 18). UT moderators represent a potentially valuable avenue to explore in the context of medical education, as these moderators could spur curricular innovations designed to support medical students to develop UT needed for their future practice (4, 19). As yet, however, research aiming to understand UT moderators within this context has yielded somewhat limited insights, which may be partly due to the research methods heretofore engaged.

Historically, there was a reliance on UT scales to study moderators (11). These studies typically focused on students' demographic factors and training stage as potential moderators, yielding rather inconsistent results (11). For example, more advanced stages of training were found to be associated with lower UT (20, 21), higher UT (21–24), as well as no significant differences in UT (13, 25). Results regarding age and gender as moderators of medical student UT are also inconsistent (11). A recent meta-analysis of UT scale reliability indicated significantly lower reliability among populations of medical students compared to physicians, as well as high levels of heterogeneity in sub-analyses (26). These findings, respectively, suggest that inconsistent results pertaining to UT moderators could relate to imprecise results among medical students, and that there are likely to be moderators of UT impacting findings beyond those assessed by primary studies (26).

By contrast, qualitative studies exploring medical students' UT are beginning to build evidence for moderators related to experience, teaching practices, peer relations and reflective writing (19, 27–30). Several studies describe a shift in students' perceptions of and responses to uncertainty, from earlier absolutist views on medicine, toward an acceptance of uncertainty as a feature of clinical practice as training progresses. This suggests that gaining experience as a medical student may moderate UT, although studies are limited to preclinical contexts (19, 27, 28).

Within the context of preclinical anatomy education, our prior research identified UT moderators pertaining to teaching practices and peer relations (19). Educators who acknowledged the presence of uncertainty and outlined the evidence-base explaining multiple possible answers were described as facilitating students' UT, whereas educators who engaged in didactic approaches that failed to address subject matter uncertainties were described as impeding UT (19). Relating to peer relations, working within a team wherein students were able to share responsibility for their uncertainty was described as aiding students' UT (19). Thus even within the context of anatomy, which is often perceived in certain terms (27, 28), educational approaches were described as moderating and developing students' UT (19). Within the clinical context, however, there is limited knowledge of moderators of medical students' UT. A study by Nevailainen et al. (29) with medical students in their first clinical year aimed to explore students' experiences of uncertainty and how these developed over time. Although this study did not purposefully aim to identify UT moderators, the authors did note that the reflective writing students participated in as part of study data collection may be beneficial in supporting students to cope with uncertainty.

As such UT research is yet to purposefully explore the breadth of moderators as experienced by medical students within the clinical education context. Identifying UT moderators may help pave the way to develop educational interventions that better prepare medical graduates for the uncertainties implicit in modern and future clinical practice, and help mitigate the negative impacts that lower UT may have for medical students and physicians, and the patients in their care. Therefore, we asked “*What factors do medical students in their clinical years perceive as moderating their perceptions of, and responses to, uncertainty?”*

## Methods

### Study Design

**Figure 1:**
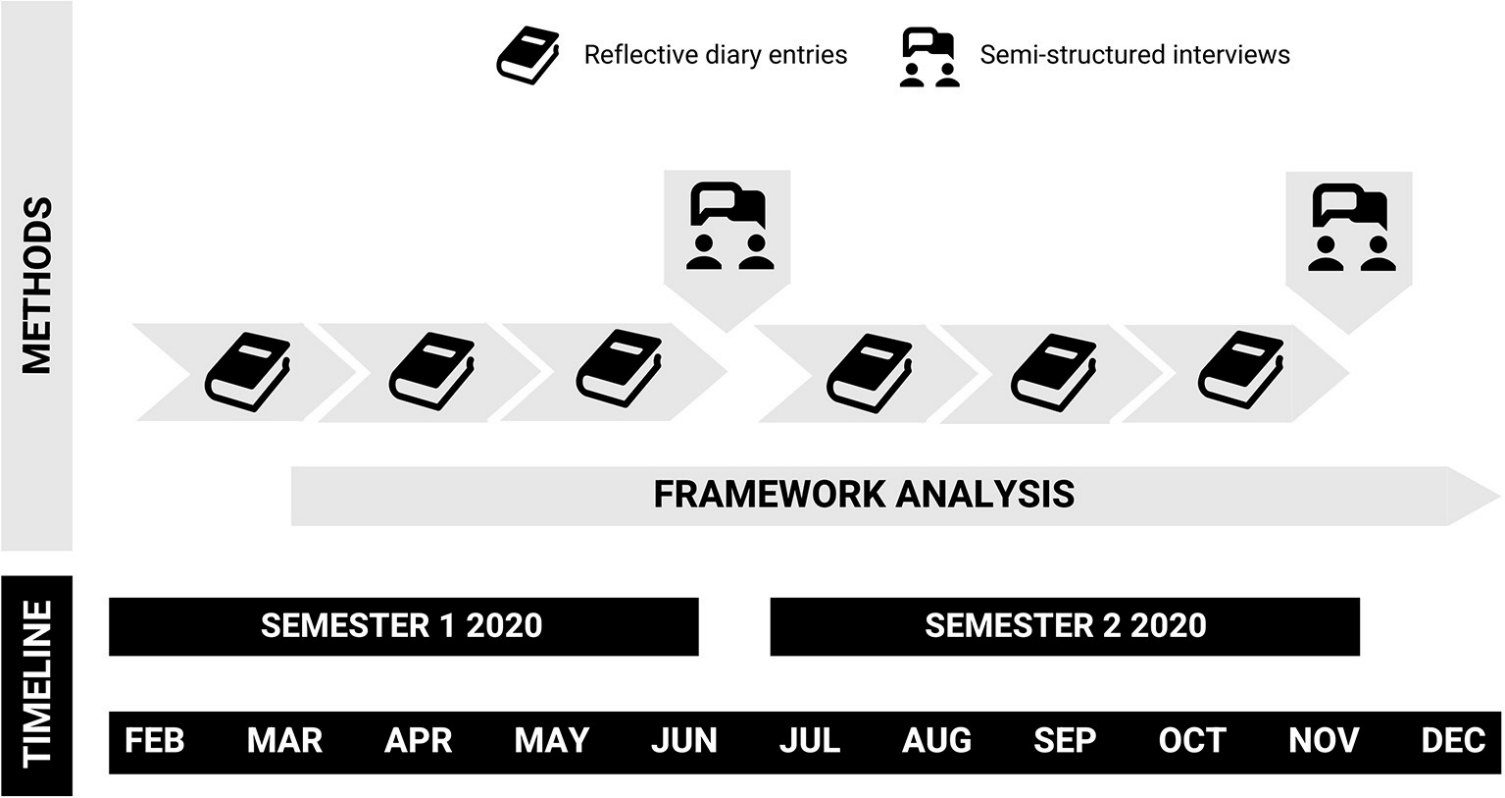
Overview of study methods and timeline. Participants described their experiences of uncertainty through in-semester reflective diary entries, and discussed their experiences through end of semester individual or group semi-structured interviews. Framework analysis of data began following the collection of the first diary entry, and continued throughout the remainder of the study.

This study, focused on UT moderators, forms part of a larger research project exploring clinical years medical students' experiences of uncertainty (31). Engaging a social constructionist paradigm (32, 33), we undertook a qualitative, longitudinal research (34–36) project at an Australian medical school. Data collection methods included participants completing both reflective diary entries, and semi-structured interviews (individual and/or group) about their experiences of uncertainty throughout the 2020 academic year (Figure 1). Methods specific to the present study are detailed here. Further details including the full semi-structured interview protocol are described in Stephens et al. (31).

### Context

The medical course at the study institution provides entry pathways for students both directly following secondary schooling (“direct entry”), and after completion of an undergraduate degree (“graduate entry”). Both entry streams accept students enrolling domestically and internationally. Although direct and graduate entry students are separated for an initial preclinical phase, the streams combine for the penultimate 3 years of the course constituting the clinical phase. The location of students' clinical placement sites includes metropolitan, suburban, regional and rural areas, ranging from primary to quaternary care settings. All students graduate with a Bachelor of Medical Science and Doctor of Medicine (MD).

### Sampling and Recruitment

For the present study, students were purposively sampled from the first (year “3B”) and final (year “5D”) years of the clinical phase, during which students are primarily placed within hospital settings. Our prior research exploring UT among preclinical students identified that transitions in education are a key stimulus for uncertainty (19). Students in year 3B are in the midst of a transition from campus-based learning to learning within the healthcare context, whereas students in year 5D are preparing for the transition to practice. We, therefore, anticipated that the transitional contexts experienced by these year levels may be particularly favorable for researching UT moderators.

Following institutional ethics approval (Project ID 20933), we recruited participants through messages disseminated via the course learning management system. In addition, G.C.S. conducted in-person and Zoom (version 5, Zoom Video Communications, San Jose, CA, USA) recruitment drives. A total of 41 students were recruited (23 in year 3B, 18 in year 5D), among which 35 completed all eight stages of the study (see “Data Collection”). Following completion of study consent, participants answered a brief demographics survey. This revealed that the majority of participants identified as women (68%), were in the direct entry stream (85%), and enrolled domestically (83%). The mean age of commencement of medical school was 19.1 years. A small majority of students identified as non-religious (56%), and the dominant ancestral groups identified were East Asian (41%), European (24%) and South Asian (22%). Participant demographics were thus generally reflective of the broader student cohort at the study institution.

#### Information Power

Using the concept of information power (37, 38), we explored whether we achieved appropriate sampling to answer our research question. We considered our sample size adequate to achieve information power for several reasons (37, 38). Firstly, our study aim was relatively narrow, focusing on UT moderators. Secondly, our population of clinical years medical students was specific to our research question. Thirdly, our data analysis was informed by existing theory on UT (3). Finally, we had a rich interviewer-participant dialogue that developed over time.

### Data Collection

#### Relationship Between Researchers and Researched

G.C.S. led communication with participants across all stages of data collection, and made herself known to participants in her role as a medical education doctoral candidate. M.D.L. had previously taught and assessed many participants in their preclinical anatomy education. As this could represent a power imbalance potentially impacting how students present themselves to a former teacher, all data were de-identified prior to analysis by M.D.L (as well as M.S.). None of the authors were involved in teaching or assessment of students during or following the study period.

#### Reflective Diary Entries

Participants were asked to complete a minimum of six reflective diary entries during the study. These were spaced such that participants submitted their diaries 3 times per semester at approximately six-week intervals (Figure 1). Participants were provided with three options for diary format (audio recorded, typed or handwritten) to facilitate a range of reflective preferences (39). Prompts for the diaries were purposefully broad. Students were asked to describe scenarios from clinical placement, or experiences as a medical student more generally, in which they felt 1. Uncertain and 2. Certain. The “uncertain” prompt was designed to solicit data pertaining to UT, whereas the “certain” prompt was included to capture further contextual details about how students perceived certainty versus uncertainty, and the factors moderating these perceptions. Preliminary data analysis identified that some students described their chosen scenarios without further reflection, which we addressed by seeking clarification from students regarding omitted details (compliant with ethics). As such a brief framework for reflection (the “*What? So what? Now what?”* approach (40)) was provided to students at the start of the second diary period, although it was emphasized that this recommendation was only a suggestion of one style in which to structure a reflection. At the conclusion of the study, participants had completed a total of 230 diary entries, representing 178,308 words. Of these, 170 were typed, 50 audio-recorded and 10 hand written. The shortest diary was 188 words, and the longest 5,119, with a mean of 775 words per diary. A summary of diary entries according to type, year level and gender of participants is provided in Table 1.

**Table 1:** Number of diary entries submitted according to year level, diary type and participant gender.

**Year level**	**Diary type**	**Participant gender**	**Number of entries**
3B	Typed	Woman	64
		Man	11
	Handwritten	Woman	8
		Man	0
	Audio	Woman	30
		Man	17
5D	Typed	Woman	55
		Man	40
	Handwritten	Woman	2
		Man	0
	Audio	Woman	3
		Man	0

#### Individual and Group Interviews

At the end of each semester, students had the option of participating in either an individual or group interview according to their preference and/or availability. Both approaches were engaged to further explore potential moderators that were identified through preliminary analysis of diary entries, facilitating crystallization of our findings (38). This dual approach of individual and group interviews was used for methodological and practical (i.e. scheduling and participant availability) reasons. Individual interviews facilitated in-depth responses and understanding of individual perspectives, whereas group interviews allowed participants to interactively share, compare and contrast their experiences with those of their peers (41). Both approaches utilized the same semi-structured interview protocol (31), and were all facilitated by G.C.S by Zoom due to social distancing restrictions in effect during the study period, as well as the geographical distance between participants' placement sites. Following our experiences in other research projects using Zoom, group interviews had a maximum of four participants, as we found that engagement was difficult to maintain with larger groups.

Interview questions were developed following early stages of the iterative analysis of diary data to ensure that we more deeply explored participants' perspectives of UT moderators, and that our developing codebook (see below) was reflective of these. Participants were first asked “*In your experiences of uncertainty, are there any factors that have impacted your experience of uncertainty either positively or negatively? These might be to do with the people involved, the setting or features of the situation itself, but could be anything you can think of that has changed your experience of uncertainty in some way.”* Further questions were guided by participants' responses, with specific prompts added about potential moderators identified in participants' diaries where required. These prompts related to potential moderators included the influence of experience, personal characteristics, other people, assessments, and approaches to teaching and learning (including reflective learning) (31). The protocol included deidentified quotations from diaries to spark further discussion as needed, however these were infrequently required.

Ultimately, we completed 20 individual interviews (10 per semester, ranging in length between 32 min and 1 h 24 min) and 20 group interviews (10 per semester, ranging in length between 55 min and 1 h 28 min). Average duration by interview type, student year level and timepoint are provided in Table 2, with a further breakdown of each interview and demographics of participants provided in Supplementary Material 1. Together, the interviews resulted in 414,708 words of data or approximately 42 h of recordings.

**Table 2:** Average interview duration according to study time point, year level of participants, and type (individual or group).

**Time point**	**Year level**	**Type**	**Number**	**Average duration**
1	3B	Individual	3	00:52:53
		Group	6	01:05:44
	5D	Individual	7	00:48:10
		Group	4	01:02:43
2	3B	Individual	5	01:03:23
		Group	6	01:23:02
	5D	Individual	5	00:54:41
		Group	4	01:21:16

*The number of interviews in each category are also provided. Time point 1 corresponds to the end of semester 1, and timepoint 2 to the end of semester 2*.

### Data Analysis

We analyzed all data using reflective, team-based framework analysis (42). Our analysis was undertaken with the Hillen et al. (3) integrative model as our initial conceptual framework, and was abductive in nature (43). Herein we oscillated between deductive (i.e., applying the integrative model to our dataset to aid our understanding of participant UT) and inductive approaches (i.e., building theory on UT moderators within the context of clinical years medical students. Framework analysis involves five steps: 1. Familiarization, 2. Identifying a thematic framework, 3. Indexing, 4. Charting and 5. Mapping and interpretation. Familiarization commenced with receipt of the first round of diary entries, and was revisited at each stage of data collection. All diary entries were read or listened to by G.C.S., with a subset of diaries (about three per time point) and interview transcripts also read by M.S. and M.D.L. Each author noted their initial impressions, which were shared and discussed at fortnightly (G.C.S. and M.D.L.) or monthly (all authors) meetings. Stage 2 commenced with G.C.S. drafting a codebook with preliminary theme names, definitions and illustrative quotations. Multiple drafts and revisions of the codebook were reviewed and edited by all authors. Stage 3 then involved G.C.S. coding the entirety of the dataset, using NVivo (version 12; QSR International, Melbourne, Australia). Progress and challenges with coding were discussed in regular meetings (all authors), with further refinements to the thematic framework agreed to when needed. Charting then involved all authors discussing the data as themes, and making further refinements to the thematic structure. Finally, mapping and interpretation involved exploring patterns in thematic dominance, linking themes to our research question, and comparing our findings with existing research. This step was finalized through the process of writing and editing the results and discussion sections of the present paper.

### Team Reflexivity

Following establishment of the research team, we engaged in a team reflexivity exercise (44). This enabled us to understand each other, and our orientations toward the proposed research. Although we all identified as social constructionist researchers, we were diverse in regard to gender, career discipline and stage, and prior experience researching UT and medical students. By way of background, G.C.S. is a graduate of the same medical school as the present study and was undertaking the present research as part of her doctoral studies, M.S. is an education researcher with a background in science education, and M.D.L is an anatomy educator and medical education researcher who originally trained as a cell biologist. By understanding each other's backgrounds and how this shaped our knowledge and beliefs about UT, we were able to challenge each other's assumptions about UT moderators throughout the process of data analysis. Thus, our reflexivity continued throughout the research, and helped ensure the rigor of our data analysis.

## Results

An overview of the thematic structure of our results is provided in Table 3. We identified four broad moderator themes described by participants: (1) Individual factors (2) Sociocultural factors, (3) Academic factors, and (4) Reflective learning. Each of these themes is described in association with illustrative quotations from participants, for whom we have designated pseudonyms for the purposes of identity protection.

**Table 3:** Overview of the thematic structure of results.

**Uncertainty tolerance (UT) moderators**	
**Theme**	**Subtheme**
1. Individual factors	a) Experience
	b) Personal characteristics
	c) Sense of purpose
	d) Social comparison
2. Sociocultural factors	a) Teaching behaviors
	b) Placement inclusivity
	c) Healthcare professional cultures
3. Academic factors	a) Assessment
	b) Orientation
	c) Faculty communication
4. Reflective learning	/

### Individual Factors

These were self-identified factors which clinical-years medical students described as influencing their UT. Subthemes encompassed students' (a) experiences, (b) personal characteristics, (c) sense of purpose, and (d) social comparison.

Participants described their medical student *experience* as having a dominant influence on their UT, with the accumulation of clinical experience acting as an aid to navigating future experiences of uncertainty:

“*[Uncertainty in medicine] gets easier with time and experience. And as students, we've only had…three years of clinical experiences…whereas…most of the doctors have had like, decades of it. And they still experience uncertainty, but it's that [clinical] experience and past experiences, which help and guide us the most.” Nisha, Year 5D, Group Interview*.

Experience was described both in terms of gaining experience with medicine and healthcare in general, as well as in relation to practicing specific clinical skills. Although experience in general was described as facilitating students' UT, the moderating influence of specific clinical skills-based experiences varied according to students' perceptions. For some, practicing skills could facilitate UT development. Alternatively, experiences of inconsistent performance seemed to hinder some students' development of UT. A typical example was “*failing”* to complete a procedural skill despite prior success:

“*One scenario in which I felt uncertain was my inconsistency in being able to cannulate patients. I had ended the previous rotation able to do straightforward cannulations in patients with good veins. The patients I encountered on this rotation however often had worse veins that were not as easy to cannulate... It was disheartening for me to come off my previous rotation with some confidence in my ability to cannulate and suddenly missing almost every cannulation I attempted.” Pallavi, Year 5D, Diary*.

This negative experience was sometimes described alongside hesitation in approaching skill development opportunities in the future, whereas other students perceived similar failures as inherent to the learning process:

“*Even when say you're uncertain in doing the procedure, and it doesn't end up working in the end, you know that you've walked away with more experience and most of the time your supervisor will give you some tips so that you're more prepared for your next experience.” Alice, Year 5D, Group Interview*.

In this way, experience as a moderator of UT is conceptualized not only in quantitative terms, but is also related to the qualitative nature of an individual's particular experiences and their perceptions of outcomes.

*Personal characteristics* students described in their diary entries and interviews as influencing their UT included demographic, personality traits, and mental health factors. Students described a wide array of demographic factors moderating their UT, including their living arrangements, employment status, relationship status, medical school enrolment intermissions, and the location of their familial home in relation to the study institution. Amongst these factors, some had a variable influence on students' UT depending on individual student perceptions (e.g. living with other students or alone, employed or not), whereas others were described exclusively as hindering UT (e.g. location of the students' familial home outside the state of the study institution, being in a relationship, and having intermitted during medical school). For example, living alongside fellow students was described as facilitating UT through sharing and learning from others' experiences, but was also described as enabling the negative aspects of *social comparison* (further described below):

“*But now that I'm like, literally in the same house, it's much harder to distance myself… I'm sort of force fed, like, my friends' study habits, I don't want to see them but like, I have no choice but to see them and like when you see them, you have no choice…to like, compare yourself…it's hard to sort of figure out where you should be sitting in the middle of that…so that's definitely been something I'm uncertain of.” Toby, Year 3B, Group Interview*.

Personality traits and mental health factors were described only in relation to negative impacts on UT with students specifically describing the influence of burnout, introversion and anxiety on their management of uncertainty:

“*I tend to be of a more anxious personality type. So, when I feel those emotions [related to uncertainty], I tend to think of them more as like my anxiety coming up…it's almost like I start to panic…I really want to get out of this situation…And my thoughts just basically start to go everywhere, in every direction. It doesn't make sense, it's very irrational…it's just a whole whirlwind of like, anxiety type emotions.” Violet, Year 3B, Group Interview*.

In contrast to negative impact of these personality factors, students described that possessing and fulfilling a *sense of purpose* facilitated UT by motivating them to navigate uncertainties:

“*I think having purpose definitely is a beneficial factor for…dealing with uncertainty… it reminds you of the bigger picture that if…you are feeling uncertain, your purpose reminds you …that you're really here for a bigger reason.” Nisha, Year 5D, Group Interview*.

Students described finding their purpose in a variety of ways, including focusing on their career path in medicine, identifying the need to learn to care for their future patients, being helpful to their clinical team, acting in patients' best wishes, and maintaining their personal values within the context of healthcare. Purpose relating to personal values included discussion of social justice issues (particularly equity in healthcare access) and values rooted in religious beliefs, with these values often underscoring students' motivation to study medicine. Maintaining these values was described as being able to provide a sense of guidance and certainty for students despite the uncertainty surrounding them:

“*I actually am having quite a bit of difficulty identifying moments or scenarios during which I felt certain. However, I do feel certain about my passion for women's health and my values rooted in intersectional feminism. I am grateful to have the opportunity to speak with women on a daily basis and be privileged enough to hear their stories and it was during those times of GP [general practice], when I was truly connecting with patients, that I felt the most certain… When I feel this way decision making is much easier and my overall confidence is increased.” Emily, Year 5D, Diary*.

The final individual student factor reported as impacting students' UT was that of *social comparison*, or the process of thinking about others in relation to oneself as a means of making sense of one's current or future position (45). This was chiefly described in relation to one's peers and near-peers. Whilst this could facilitate UT for some participants (e.g. adopting uncertainty management strategies observed in others), this subtheme predominately appeared to hinder UT. Here participants focused on differences with peers whom they perceived as having access to advantageous learning opportunities (e.g. placed at clinical sites believed to have a better quality of education), or who appeared to outperform them academically. This could lead to students doubting their own learning abilities:

“*One of the students in my group, he is absolutely amazing…And it is really hard to not to compare myself to him. So, I'm like, wow, I am next to this amazing person who knows so much more. Why don't I know that much? What am I doing wrong?” Bianca, Year 3B, Individual Interview*.

In this way, social comparison could amplify students' uncertainties about learning, and sow doubt about their abilities to become a successful doctor in the future.

### Sociocultural Factors

Sociocultural factors described by students as influencing their uncertainty were (a) teaching behaviors, (b) placement inclusivity, and (c) healthcare professional cultures.

*Teaching behaviors* were described as either facilitating or hindering students' UT depending on the educators' approach. Medical educators were identified by students as spanning the continuum of career stages from near-peer medical students to senior clinicians. Teaching behaviors described as facilitating UT included role-modeling UT, scaffolding knowledge (including selective didactic teaching), encouragement, constructive feedback, acceptance of mistakes and setting learning goals and expectations. For example, acceptance of mistakes seemed to facilitate UT by allowing students to distance themselves from negative emotions associated with uncertainty, and refocus their attention toward uncertainty as part of the learning process, whereas setting learning expectations provided students with a framework for managing learning uncertainties:

“*I did have a clinical bedside tutor and in their first session with us…they were just like, ‘okay, I really want to set expectations of how I want this teaching to go… that this is a space where you can make mistakes… I want to figure out…what point in your journey you're at, because I'm a consultant and I'm so far from that now.' So, I think that's positive because it … gave us some certainty around expectations and also how to navigate the times when we were uncertain.” Leena, Year 3B, Group Interview*.

Role-modeling UT was described through educators exposing their thought processes, dilemmas and failures [i.e. intellectual candor (46)] about uncertainty in medicine. Intellectual candor can be used to invite reciprocal vulnerability from students', build trust, and drive learning within professional settings (46). Within the present study, intellectual candor seemed to allow students to appreciate the inherent uncertainties of medical practice, and build confidence that they can and will learn to manage these:

“*[The surgical registrar] was asking me why he wasn't cutting here … I said, ‘I didn't know' and he was like ‘…it's because there's a chance of cutting the inferior epigastric [artery] if you cut here…Ask me why I know that?' And I was like, in my head…'Oh, so you're going to tell me 'I knew this when I was a third year' or something', and he was like, 'Oh no, when I was reg somewhere else, I actually cut it and since then, I've made sure that I don't.' And so, I kind of felt like him offering that to me was actually like, look, I didn't know as well and I paid a price and that's why I'm teaching you this…I think for him to actually like reveal a personal flaw or maybe something like that was actually quite generous, but also made me feel like oh okay, everyone doesn't know things.” Cathy, Year 5D, Individual Interview*.

Despite the many positive teaching behaviors described, students also experienced a variety of behaviors perceived as hindering their UT. These behaviors included singling out students from a group to answer questions, didactic teaching as a standalone pedagogical approach, acts of learner humiliation, false assumptions about students' prior learning, and inadequate supervision:

“*One of the probably biggest factors [that impact uncertainty] is… supervision. So, the current rotation…it's a very busy rotation...I found…the busier the people in the team you're with, the less time they have to direct you in the right way. So, then you show up uncertain and if there's no one there to kind of guide you because they're extremely busy, that just…kind of snowballs it.” Christopher, Year 5D, Individual Interview*.

When analyzing teaching behavior patterns according to the educator's career stage, we identified that positive behaviors were described in relation to teaching by near-peer medical students, junior doctors and senior doctors. However, teaching behaviors described as hindering UT were more typically described in relation to senior doctors. Students described the cognitive and social congruence (47) of near-peers and junior doctors as reasons why these educators could be of particular assistance to navigating uncertainties:

“*I think, a…really big factor that has helped alleviate uncertainty for me have been my [year 5D student] mentors…I think just having these senior mentors…who know their way around and have been in our shoes really helps, and the fact that they're kind of closer in age and in…their journey to us compared to like, big consultants, really helps them like relate to us.” Linda, Year 3B, Group Interview*.

*Placement inclusivity* encompassed whether students perceived their experiences as shared or isolated (typically in relation to their peers), and whether they felt included or excluded from their clinical team on placement (typically in relation to junior doctors). Inclusion and active involvement within a clinical team was described as facilitating UT, and could be achieved through simply addressing students by name and acknowledging their presence on ward rounds within the medical record:

“*The HMO [house medical officer] would put my last name next to theirs in the patient notes, and would introduce me to the patient by name. It was such a simple thing to do but it made me immensely more comfortable in learning from them and asking questions – without feeling like a burden or like I was unwanted.” Ainsley, Year 3B, Diary*.

Conversely, exclusion from the clinical team included instances of students being ignored and/or actively excluded from typical placement opportunities such as ward rounds. This served to amplify students' uncertainty about learning on placement, as they felt they too much of a “*burden”* on their clinical team to discuss uncertainties.

Whereas placement inclusivity centered on the students' perceptions related to small teams of individuals encountered on placement, *health professional cultures* more broadly described the customary behaviors of different health professional groups that students encountered. Specific cultural aspects described by students influencing UT were the hierarchies within medicine, and the tribalism between different healthcare professional microcultures. Medical students' perceived inferior hierarchical status within medicine could compound their uncertainties, wherein students described questioning their own knowledge or perceptions when these differed to that of a senior:

“*It's sort of come down to…me sort of like questioning people who are senior to me…There was another instance the other day where our [general surgery registrar] went into a patient's room and called the patient one name when it was a different name on the list… he went through the whole consult, telling this patient that … he had multiple [pulmonary embolisms]…he only noticed right at the end, that he was actually talking to the wrong patient…so I'm like, maybe I'm the one who's wrong… maybe it's the list…that's wrong, because obviously this the reg seems really confident.” Aarush, Year 5D, Group Interview*.

In healthcare tribalism, the differing beliefs and values held by different healthcare professional groups (e.g. physicians versus surgeons, doctors versus nurses) also served to compound students' uncertainty about learning within the relatively unstructured clinical placement context:

“*Our tutor has also given instructions such as ‘don't ask the nurses as they overprotect their patients', which makes it more difficult when no doctors seem to be free or around for us to speak to.” Linda, Year 3B, Diary*.

Concerningly, a culture perpetuating the discrimination of minority and marginalized people was described by some students, with this influencing some students' UT. Descriptions included students' observations of healthcare professional-patient interactions, as well as students' own experiences as subjects of discrimination. These “*vulnerable”* patient and student groups thus had to contend with navigating the compounded uncertainties of institutions and culture constructed by and for those with greater privileges:

“*It's mostly been sort of an issue of racism…that I've experienced…Sticking up for myself …[is] something that definitely I do not feel comfortable doing, that I definitely feel uncertain about doing in the hospital environment…There was an instance on the wards the other day that a patient was… unintentionally kind of racist...and the reg played it off…I kind of had to just bite my lip and not say anything…there's even been instances where… I've been called sort of racist terms by staff in the hospital as well…it doesn't feel nice not being able to stand up for yourself in the hospital environment.” Aarush, Year 5D, Group Interview*.

A culture of social inclusion facilitating UT (i.e. theoretical converse of culture perpetuating the discrimination of minority and marginalized people) was not identified within the data.

### Academic Factors

Academic factors pertained to moderators enacted at the level of medical school programs and their administration. Moderators described by students included (a) assessment, (b) orientation and (c) faculty communication.

The influence of *assessment* on UT was divisive for students. Some students described assessments as facilitating UT by providing structure to guide them through learning uncertainties, whereas others felt the objective nature of assessments impeded their ability to engage with the uncertainties of clinical medicine. Indeed, the lack of summative assessments in Year 5D was described by some students as facilitating engagement with clinical uncertainty:

“*Without the stress of exams looming over our heads…I think I am finally enjoying the “art of medicine” (where previously I would hyper focus on the “science of medicine” because exams). This year, I have found that I better embrace the ambiguous [and] uncertain situations as I am less driven to be learning just for the sake of an assessment.” Chara, Year 5D, Diary*.

Provision of an *orientation* to clinical placements appeared to vary between clinical sites. When formal placement orientation was provided, this was described as reducing the perceptions of uncertainty related to learning within unfamiliar placement contexts, and facilitating capacity to manage uncertainties related to learning clinical medicine:

“*What [our placement administrator] tried to do is… give us a tour of the [emergency department] to like help orientate us and little things like that have taken away a little bit of the uncertainty…And they sent a whole document at the start of the rotation talking about the different roles of the different areas and what the roster is. And they really helped to take away a lot of the uncertainty and so now it's…I think it's just the right amount of uncertainty now… where you feel like you have that space to grow and that space to learn.” Olivia, Year 5D, Group Interview*.

When formal orientations were omitted, some students described initiating their own peer-to-peer orientations, including handover document development and communication via group messaging applications. This self-directed approach to orientation also appeared to facilitate UT, as students managed their placement uncertainties by sourcing information from peers who had previously completed the same placement.

Students described that the style of *communication* from those in the faculty (i.e. medical school and clinical site leaders and administrators), and whether this was perceived as supportive of students, served to moderate their UT. This sub-theme was particularly described within the context of pandemic-related impacts to placements. Knowing that they were supported by the faculty seemed to facilitate students' UT, even in the face of significant and enduring uncertainties. A cornerstone indicative of support was frequent communication interpreted as conveying the primacy of students' educational interests:

“*To have faculty support and to know that they had our backs despite all the uncertainty was very reassuring, even as I understood that the situation was very fluid. This encouraged me and allowed me to feel supported and reassured on an academic level, and ultimately gave me enough peace to make difficult decisions.” Patrick, Year 5D, Diary*.

By contrast, infrequent, untimely and conflicting communication from faculty was perceived as unsupportive of students, and could magnify students' uncertainties about possible disruptions to learning and assessments. This was especially the case when communication from faculty differed from messages students received from other health professional staff working at students' placement sites:

“*There is a lot of confusion, in the sense that directives we as students are receiving are quite mixed. [The university] has standardized it recently, so that the clinical school you are associated with dictates things, however, the head nurse, the consultant, registrar and other staff that you work with do provide directives…[that]…can be mixed.” Ali, Year 5D, Diary*.

### Reflective Learning

Although the primary role for the research diaries was to explore clinical students' experiences of uncertainty, the way in which participants described their experiences suggested that the reflective process was, itself, moderating UT. The role of reflection as a moderator was further supported by students through their interview responses. Reflections appeared to influence students' perceptions about past uncertainties, as well as future uncertain scenarios. Dominant reflections described by students involved “*reframing”* uncertain scenarios by taking the focus away from negative associations with uncertainty, and instead focusing on ways in which uncertainty could be beneficial:

“*I kind of enjoyed being able to write out the diary entries. It's sort of forced me to look back on things and look back on what I've gained…from a rotation, or what I haven't gained and need to carry into the next rotation…So… I thought it's actually been beneficial for me as well to be able to reflect on that uncertainty and really think about it in, you know, in terms of uncertainty as opposed to just failings.” Pallavi, Year 5D, Individual interview*.

The predominant way in which students reflected on uncertainty was in terms of identifying learning opportunities facilitated by uncertainty. Students recognized that although uncertainty could be uncomfortable, it was inherent to learning and the practice of medicine:

“*Uncertainty is quintessential to our learning now, as it pushes us to learn and find out things. I mean, the only way to learn is to not know, and I want to be both comfortable in not knowing, while still wanting to reduce the amount of things I don't know.” Harrison, Year 3B, Diary*.

Reflections on uncertainty also discussed identifying personal growth and increasing the capacity to take appropriate steps in the face of future uncertainty. Students described that contending with uncertainties developed their confidence and resilience within healthcare contexts:

“*I think I have become more resilient throughout the year… So, when I was bit uncertain about things, or felt a bit kind of insecure in a moment, I would kind of regroup, think and just continue on, or come up with a plan B.” Natalie, Year 3B, Individual Interview*.

There was, however, a darker side to reflection described by some students, including rumination and a sense of regret. Ruminating on uncertainty involved a persistence or amplification of negative responses to uncertainty that students recalled feeling in the moment of uncertainty:

“*I feel like sometimes my uncertainty gets, like, amplified because I have a tendency to like, overthink things, like after the situation and like, ruminate about things. So, yeah, in some ways, I feel like it, I've become more uncertain, like the further it becomes from [the uncertain situation].” Victoria, Year 5D, Group Interview*.

Students who described regret on reflection seemed to perceive their uncertainty in terms of inadequacies or subpar performance in a learning encounter. Unlike positive reflections, this regret was not countered with a sense that the uncertain experience could be of benefit in some way:

“*I definitely beat myself up a bit if the outcome isn't necessarily good, because all you can think is I should have done better. I knew the answer to that, or I know what I should have done in that scenario. Why do I know that now? Why didn't I think about that 5 minutes ago? All those sorts of feelings.” Raimon, Year 3B, Individual Interview*.

These negative reflections were less typically described by students, with the dominant pattern we identified being that reflective learning facilitated UT. Furthermore, this facilitation could occur despite recalling negative responses (e.g. worried, overwhelmed) experienced at the time of students' initial uncertainty.

## Discussion

We purposefully explored UT moderators as perceived by medical students in their clinical years. In doing so, we identified a broad range of moderators, encompassing *individual factors, sociocultural factors, academic factors*, and *reflective learning*. Our findings both refine and extend the Hillen et al. (3) model within the context of clinical medical students. Hillen et al. (3) list the moderator categories of (a) stimulus characteristics, (b) individual characteristics, (c) situational characteristics, (d) cultural factors and (e) social factors, but have otherwise not defined nor further described these terms. We chose to use the term *individual factors* instead of individual characteristics to imply a broader range of moderators within this theme, as the individual factors we identified encompassed both demographic characteristics, and other facets of students' experiences and character. Within our data and the real-world healthcare environment perceived by students, separating social and cultural factors was difficult, thus we combined these within *sociocultural factors. Academic factors* is perhaps a more specific moderator of relevance to our study context, but may incorporate aspects of situational or stimulus characteristics from Hillen et al. (3).

*Reflective learning* does not clearly align with any of the Hillen et al. (3) model categories, and thus constitutes an extension to the existing model. This moderator represents an important potential avenue for moderating UT in the medical student context, as reflective skills can likely be developed through educational interventions. Based on their work within the context of medical students in their first clinical year, Nevailainen et al. (29) describe that reflective writing may be a “powerful tool” for students' professional development as it concerns uncertainty. Our findings further extend this by supporting the role of *reflective learning* across written as well as audio reflections, thereby providing flexible options for students.

Amongst our identified moderators and their subthemes, many were described as having a variable influence on students' UT, either facilitating or hindering UT depending on specific student perceptions and contexts. This differs from existing research that typically only describes moderators in terms of being associated with one or the other of higher or lower measured UT. For example, prior research typically describes experience as a moderator in terms of quantifiable experience gained (e.g. number of years in practice), and demonstrated inconsistent results regarding whether UT increased or decreased with experience (11). In our data, experience could either facilitate or hinder UT, depending on an individuals' perceptions. Notably, an experience perceived negatively (e.g. “*failure”*) could hinder UT, effectively trumping previous experience gained. Thus, describing experience only in black-and-white quantifiable terms is likely insufficient to fully understand this moderator, as our research suggests that the nature of these experiences additionally influences UT.

Although our research supports UT as a modifiable state, questions do remain regarding the possibility of a trait level component to UT (i.e. a relatively stable and consistent pattern of response to uncertainty over time). Inherent differences in UT traits between individuals could explain the differing perceptions students had about some moderators (e.g. assessment), and why some students tended to ruminate upon reflection. In this way, trait level UT could be conceptualized as an additional *personal characteristic* influencing students' UT, or act as a set point around which moderators are able to exert an influence. Critically, however, our data supports the notion that students perceive that their UT can be moderated with the assistance of medical educators and educational institutions.

### Suggestions for Clinical Education

**Figure 2:**
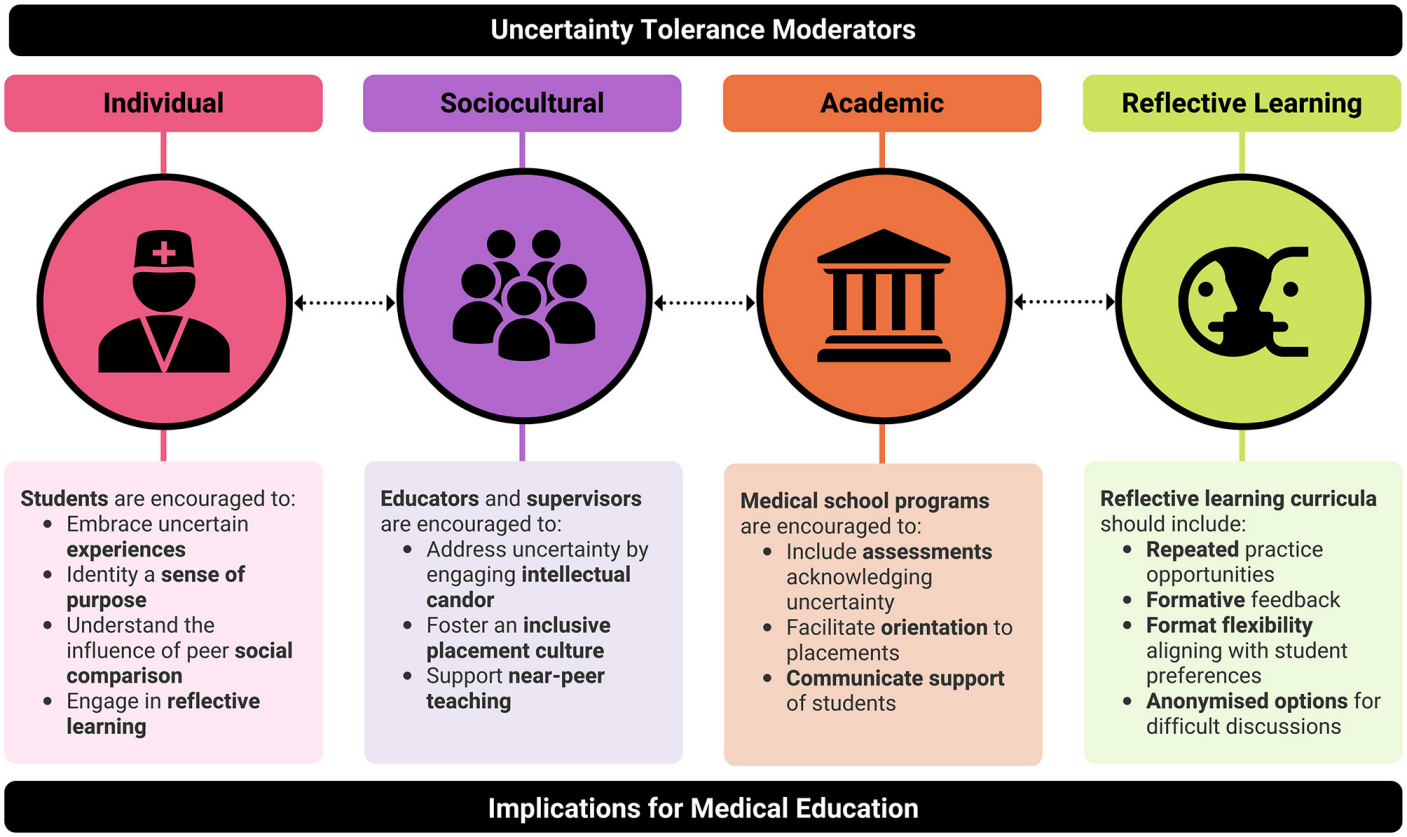
Uncertainty tolerance (UT) moderator themes and suggested translations to clinical education. Dotted arrows between themes indicate that successful development of medical students' UT should ideally involve interventions that cut across all aspects of medical education.

Many of the moderators we identified may have potential implications for clinical education (Figure 2). Crucially, *reflective learning* appears to have the potential to powerfully influence clinical students' UT in a manner that may help counter other moderators with a negative influence. For example, engaging with *reflective learning* may benefit clinical students who perceive negative experiences related to uncertainty, allowing these to be reframed into *learning opportunities*. The clinical students in our study valued the repeated, formative nature of the reflective diary entries, the flexibility to choose their reflective medium, and the ability to reflect on and share their experiences through interviews. More generally within medical education, reflective learning is known to assist in developing skills for lifelong self-regulated learning, and for managing the complexities of practice (39). Prior research also suggests that the benefits of reflective learning may be maximized by avoiding summative assessment of reflections, as apprehensions related to assessment may impede students' engagement in the reflective process (48). Although our research demonstrates that reflection on uncertainty was predominantly a positive experience for students, the risk of negative reflections such as rumination suggests that reflective learning should ideally include provisions for supportive/pastoral care.

The positive reflections identified in our study could serve as specific prompts for clinical students to use in a guided reflection. For example, clinical students could be asked “what did you *learn* from uncertainty in this scenario?” and “how can this experience make you more *confident* or *build resilience* for similar scenarios you may encounter in the future?” Reflecting on the role of *social comparison* and *purpose* related to uncertainty may also prove beneficial topics. As sharing reflections can be challenging and leave clinical students open to potential loss of face, one approach could be to engage the use of an anonymized discussion forum (19). Our own prior research into UT in preclinical settings revealed that such approaches facilitated preclinical student engagement with discussion topics, as well as their UT development (19).

Also related to reflection within the context of medical education, we identified that educators who practice intellectual candor (46) may also powerfully impact clinical students' UT. The examples of intellectual candor described by clinical students typically involved educators reflecting on past experiences of uncertainty, including when they were students themselves. In this way, intellectual candor may have helped to break down the perceived lack of cognitive and social congruence between clinical students and more senior clinicians. In order to build clinical students' UT, these educators then continued to discuss their thought processes regarding how they constructively managed uncertainty. Thus, the practice of intellectual candor by educators seems to develop clinical students' appreciation for the pervasiveness of uncertainty in healthcare, and build confidence that they will learn to manage these.

The variable influence of *assessment* on learner UT identified in this study raises questions about how educators can balance the benefits of assessments that drive learning, whilst minimizing the described negative impacts of assessment on UT. One possible solution warranting further research may be pass/fail grading. When compared to tiered grades, pass/fail assessments may be associated with improved student wellbeing without adverse impacts on academic performance (49). When combined with regular, formative supervisor feedback, pass/fail assessments are linked with intrinsic motivation for patient care, and a sense of learner agency (50). This approach may be ideal for balancing students' motivation for and engagement with patient care uncertainties, with sufficient feedback on their performance to guide learning. Of note, the assessments experienced by participants in this study were not specifically designed to evaluate students' skills for managing uncertainty. In future, medical educators may need to devote greater attention to incorporating issues of uncertainty within assessments. We would recommend approaches that avoid “single best answers”, and instead focus on the range of considerations needed to arrive at a preferred solution. Clinical case discussions, where the focus of the assessment is on process and reasoning, and not a single answer, may be an ideal approach to facilitate this.

Finally, discussion of *orientation* and faculty *communication* moderators highlight the role these more administrative aspects of medical education can play in helping students navigate uncertainty. Our research suggests that reducing administrative uncertainties, and communicating support of students through their uncertainties, aids students' capacity for managing uncertainty related learning clinical medicine. To ensure orientation programs appropriately address students' needs as well as build skills for managing uncertainty related to frequent rotations during postgraduate training, an ideal approach may be to combine educator supervised approaches alongside facilitation of peer-to-peer handovers.

Although our study was conducted within the context of clinical years students, many of our suggestions may also be helpful for preclinical students, especially where moderators may allow early years students to build skills in preparation for the uncertainties of clinical placements (e.g. *reflective learning*).

### Implications for Future Research

In addition to exploring and evaluating how proposed interventions may impact learner UT, future research may also turn to exploring the nuances of moderators and their reported variable impacts. Researchers may need to engage a variety of methodologies, and explore the perspectives of others involved in students' education (e.g. medical educators, clinical supervisors and faculty leaders). For example, think-aloud protocols could be used to deeply explore students' experiences of uncertainty as they engage with uncertainty stimuli, and learning analytics could provide data approximating students' actual responses to uncertainty. Further research is also needed to explore UT moderators in a wider range of medical education settings (e.g. postgraduate training, continuing professional development), and develop assessment strategies that balance evaluation of learning medical knowledge, with skills for managing the uncertainties of clinical practice.

### Study Strengths and Challenges

Key strengths of our study that helped ensure the rigor of our work include our attention to information power (37) and the relationship between researcher and researched (32), the breadth and depth of our data which facilitated the crystallization of our findings (38), the engagement of existing UT theory (3), and our reflexivity throughout the research project (51). A challenge we encountered was the pandemic context and interruptions to student placements. Although this may have limited findings related to moderators within the healthcare environment, the substantive uncertainty stimulus provided by the pandemic may have brought uncertainty to the forefront of participants' minds and indeed facilitated discussion of moderators. As the pandemic represents a globally shared stimulus of uncertainty, this may aid in the transferability of our findings to other medical schools and students experiencing similar uncertainties (52).

## Conclusions

Given the inherent ambiguity, complexity and indeterminacy of healthcare, managing uncertainty remains a critical attribute for medical graduates (1, 4). Our research identified a broad range of moderators perceived by medical students in their clinical years as influencing their UT. Critically, these moderators suggest approaches to teaching and learning that may be engaged by medical educators in order to develop students' UT. Ultimately this work highlights potential areas for exploring educational interventions that may aid in preparing medical graduates for the changeable and uncertain future of healthcare practice.

## Data Availability Statement

The datasets presented in this article are not readily available because the raw data cannot be shared outside the authorship team due to ethical restrictions. Sharing of de-identified data sets supporting coding and analysis may be shared in limited circumstances and depending on compliance with ethical approvals. Requests to access the datasets should be directed to michelle.lazarus@monash.edu.

## Ethics Statement

This study involving human participants was reviewed and approved by the Monash University Human Research Ethics Committee (MUHREC). The participants provided their written informed consent to participate in this study.

## Author Contributions

Funding for this study was obtained by ML. Participants were recruited by GS and ML. All data were collected and present article was drafted by GS. All authors contributed to the design of the study, analysis, interpretation of data, critically reviewed and edited subsequent drafts of the article, and gave their approval for the publication of the final version.

## Funding

This research was funded by a Faculty of Medicine Nursing and Health Sciences Learning and Teaching Grant.

## Conflict of Interest

The authors declare that the research was conducted in the absence of any commercial or financial relationships that could be construed as a potential conflict of interest.

## Publisher's Note

All claims expressed in this article are solely those of the authors and do not necessarily represent those of their affiliated organizations, or those of the publisher, the editors and the reviewers. Any product that may be evaluated in this article, or claim that may be made by its manufacturer, is not guaranteed or endorsed by the publisher.
