# Supplementary material for: Medical Student Experiences of Uncertainty Tolerance Moderators: A Longitudinal Qualitative Study
Source: Front Med (Lausanne). 2022 Apr 25;9:864141. doi: 10.3389/fmed.2022.864141 (PMC9083353; doi:10.3389/fmed.2022.864141)
Supplement: Supplementary file 1 [file Table_1.DOCX]

Supplementary Material

# Breakdown of semi-structured interviews according to time point, year level of participants, interview type (individual or group), participant demographics (gender and entry pathway), and interview duration. W = Woman, M = Man, D = Direct entry pathway, P = Postgraduate entry pathway.

| **Time Point** | **Year Level** | **Type** | **Reference Number** | **Participant Details** | **Duration** |
| --- | --- | --- | --- | --- | --- |
| 1 | 3B | Individual | 1 | WP | 00:50:54 |
|  |  |  | 2 | MD | 00:52:07 |
|  |  |  | 3 | WP | 00:55:38 |
|  |  | Group | 4 | MD, WD, WD | 01:04:58 |
|  |  |  | 5 | WD, WD | 01:05:53 |
|  |  |  | 6 | WD, WD, WD | 00:56:56 |
|  |  |  | 7 | WD, WD, MD, FD | 01:05:58 |
|  |  |  | 8 | MD, WD, WD, WD | 01:16:23 |
|  |  |  | 9 | WD, WD, MD, WD | 01:04:16 |
|  | 5D | Individual | 10 | MD | 00:52:56 |
|  |  |  | 11 | WP | 01:01:55 |
|  |  |  | 12 | WD | 00:49:23 |
|  |  |  | 13 | MD | 00:41:12 |
|  |  |  | 14 | MD | 00:56:46 |
|  |  |  | 15 | MP | 00:42:58 |
|  |  |  | 16 | WD | 00:32:01 |
|  |  | Group | 17 | MP, WD, MD | 01:03:27 |
|  |  |  | 18 | WD, WD | 00:55:35 |
|  |  |  | 19 | MD, WD, WP | 01:03:22 |
|  |  |  | 20 | WD, WD, MD | 01:08:26 |
| 2 | 3B | Individual | 21 | WP | 01:09:57 |
|  |  |  | 22 | WP | 00:57:38 |
|  |  |  | 23 | WD | 00:52:14 |
|  |  |  | 24 | WD | 01:23:50 |
|  |  |  | 25 | MD | 00:53:14 |
|  |  | Group | 26 | WD, WD, MD, MD | 01:27:37 |
|  |  |  | 27 | WD, WD | 01:26:11 |
|  |  |  | 28 | WD, WD | 01:00:55 |
|  |  |  | 29 | WD, WD, WD, WD | 01:27:51 |
|  |  |  | 30 | WD, MD | 01:11:26 |
|  |  |  | 31 | WD, MD, WD | 01:27:48 |
|  | 5D | Individual | 32 | MD | 00:47:16 |
|  |  |  | 33 | MP | 01:00:39 |
|  |  |  | 34 | WD | 00:39:13 |
|  |  |  | 35 | MD | 00:53:12 |
|  |  |  | 36 | WP | 01:13:07 |
|  |  | Group | 37 | MD, WP | 01:16:56 |
|  |  |  | 38 | WD, WD, MD | 01:21:37 |
|  |  |  | 39 | MD, WD, WD | 01:27:55 |
|  |  |  | 40 | WD, WD, WD, MD | 01:18:34 |
